# Analysis of the correlation between the dose exposure intensity and apatinib in advanced gastric cancer: a retrospective cohort study

**DOI:** 10.3389/fonc.2025.1470462

**Published:** 2025-02-05

**Authors:** Xiao Ma, Lan Gao, Siying Che, Chaofeng Tao

**Affiliations:** ^1^ Department of Oncology, Beijing Friendship Hospital, Capital Medical University, Beijing, China; ^2^ Department of Radiation Oncology, The Third Affiliated Hospital of Kunming Medical University/Yunnan Cancer Hospital, Kunming, Yunnan, China; ^3^ Department of Pathology, The Third Affiliated Hospital of Kunming Medical University/Yunnan Cancer Hospital, Kunming, Yunnan, China; ^4^ Department of Oncology, Panzhou People’s Hospital, Panzhou, Guizhou, China; ^5^ Department of Dentistry, The First People’s Hospital of Zhaotong, Yunnan, China

**Keywords:** apatinib, gastric cancer, dose exposure intensity, efficacy, safety

## Abstract

**Background:**

Apatinib is a small molecule anti-angiogenesis targeted drug that has demonstrated significant efficacy as a late-line treatment in advanced gastric cancer in phase 3 clinical trials. This study amid to evaluate the correlation between dose exposure intensity and efficacy and safety of apatinib in the treatment of advanced gastric cancer.

**Methods:**

We conducted an observational, retrospective cohort study of patients with advanced gastric cancer who received apatinib targeted therapy in Beijing Friendship Hospital affiliated to Capital Medical University between June 1, 2018, and June 30, 2021. Dose exposure intensity (DEI) was defined as the product of dose and continuous medication time. Patients were assigned to high-dose exposure intensity (HDEI) and low-dose exposure intensity (LDEI) cohorts. The primary endpoint was progression-free survival (PFS), and the secondary endpoints were overall survival (OS) and safety. The relationship between HDEI and LDEI and clinical outcomes was analyzed by using the Kaplan-Meier curve and χ^2^ test.

**Results:**

61 patients were enrolled and assigned into two retrospective cohorts. The median PFS (mPFS) were 6.50 months (95% confidence interval (CI) [4.80-9.20]) and 4.10 months (95% CI [3.70-5.20]), and the median OS (mOS) were 10.70 months (95% CI [9.20-18.50]) and 7.50 months (95% CI [6.80-9.30]) for the HDEI and LDEI cohorts, respectively. The mPFS were 5.85 months (95% CI [5.00-7.00]) and 4.60 months (95% CI [4.10-5.90]), and mOS were 9.60 months (95% CI [9.10-12.40]) and 7.60 months (95% CI [7.20-10.20]) the for the 250 mg cohort and 500 mg cohorts. The mPFS were 6.65 months (95% CI [5.90-9.20]) and 4.10 months (95% CI [3.90-5.20]), and the mOS were 11.20 months (95% CI [9.20-18.50]) and 7.60 months (95% CI [7.20-9.60])for the long medication time and short medication time cohorts, respectively. The most common TRAEs of any grade were hypertension, proteinuria, and neutrophil count decreased. The incidence of grade 3-4 adverse reactions in the 500 mg cohort was higher than the 250 mg cohort (P=0.0016).

**Conclusion:**

The efficacy of apatinib in advanced gastric cancer was significantly positively correlated with dose exposure intensity, and HDEI can prolong PFS and OS. Early application of low-dose apatinib (250 mg/d) can improve patients’ tolerability, and the adverse reactions are controllable.

## Introduction

The diagnosis rate of early-stage gastric cancer is low, and most gastric cancer patients have reached the mid-to-late stage when seeking medical treatment, losing the chance for surgery, with the median survival period of less than 12 months (1). First-line systemic therapy of advanced GC regimens with two cytotoxic drugs like a fluoropyrimidine and platinum agent (2). The recommended second-line treatment is a combination of paclitaxel and ramucirumab (3).At present, several targeted therapeutic agents, trastuzumab, pembrolizumab/nivolumab, and entrectinib/larotrectinib, have been approved by the FDA for use in advanced gastric cancer (AGC) (4). Although systemic chemotherapy is the main option for AGC patients, with combinations of chemotherapy, immunity, and targeting have become common treatment options for AGC.

Apatinib is a small molecule anti-angiogenesis targeted drug with clear clinical efficacy in many solid tumors (5–10). In clinical practice, the recommended standard dose of apatinib (850 mg/day) is the maximum tolerated dose for the patient. However, Serious adverse reactions often occur at this dose, including gastrointestinal bleeding, hypertensive crisis, myelosuppression, and hand-foot syndrome, leading to treatment interruption without significant clinical efficacy. Despite the lack of relevant large-sample clinical study data, the initial dose of apatinib commonly used in clinical practice is 250 mg or 500 mg (11–13).

This study explores the clinical efficacy and safety of apatinib at different dose exposure intensities by analyzing the relationship between the dose exposure intensity, single daily dose, and duration of apatinib and progression-free survival (PFS), overall survival (OS), guiding the clinical medication and individualized use of apatinib.

## Materials and methods

### Study design and participants

We conducted an observational, retrospective cohort study in patients with AGC patients taking 250 or 500 mg apatinib in Beijing Friendship Hospital affiliated to Capital Medical University between June 1, 2018, and June 30, 2021. The study was conducted in accordance with the Declarations of Helsinki and approved by the ethics committee of Beijing Friendship Hospital affiliated to Capital Medical University.

Patients with histologically confirmed advanced or metastatic gastric or gastroesophageal junction adenocarcinoma and taken 250 mg or 500 mg apatinib were eligible for enrollment. Additional enrollment criteria were as follows. Inclusion criteria: expected survival >3 months; Eastern Cooperative Oncology Group (ECOG) performance status grade of 0 to 2; patients taking anticoagulants must have an international normalized ratio(INR) <1.5 and an activated partial thromboplastin time (APTT) <1.5 times the upper limit of normal (ULN) within 7 days before treatment; at least 1 measurable lesion according to The Response Evaluation Criteria In Solid Tumors (RECIST).

Exclusion criteria: Patients have been treated with vascular endothelial growth factor receptor (VEGFR) inhibitors; uncontrolled hypertension: systolic blood pressure >150 mmHg or diastolic blood pressure >100 mmHg; liver and kidney dysfunction; according to the New York Heart Association(NYHA), patients with severe cardiovascular disease, myocardial ischemia, myocardial infarction greater than Grade II, heart function Grade III-IV, and left ventricular ejection fraction less than 50% on Doppler ultrasound; patients with medical history or examination results indicate an inherited bleeding tendency or coagulation disorder.

### Treatment

All patients received oral apatinib, 250 mg or 500 mg, once daily. Continuous administration of apatinib for four weeks constitutes one treatment cycle. Investigators assessed efficacy evaluation according to RECIST version 1.1 criterion, which needed to be confirmed every 3 cycles. Patients with the controlled diseases(complete response, partial response, or stable disease) were maintained with apatinib until disease progression, intolerable toxicity, initiation of other anticancer therapies, loss to follow-up or death. The treatment could be suspended or discontinued if significant hematological or non-hematological toxicity occurred.

### Cohort

Dose exposure was defined as the product of dose and continuous medication time. Those with dose exposure intensity higher than the average dose exposure intensity were included in the high-dose exposure intensity (HDEI) cohort, and those lower than the average dose exposure intensity were included in the low-dose exposure intensity (LDEI) cohort. Patients taking 500 mg apatinib were included in the high-dose cohort, and patients taking 250mg apatinib were included in the low-dose cohort. The medication time is the sum of the time of apatinib use. Those with medication time longer than the average medication time were included in the long medication time cohort, and those with shorter than the average medication time were included in the short medication time cohort.

### Endpoint

The primary endpoint was PFS defined as the time from the enrollment date to the date of first documented disease progression or death. The secondary endpoint included OS and safety. The OS is defined as the time from the enrollment date to the date of death from any cause. All adverse events were graded according to the National Cancer Institute Common Terminology Criteria for Adverse Events (NCI-CTCAE) version 5.0 (14).

### Statistical analysis

Statistical analysis used R language. Continuous variables with a normal distribution were described using mean ± standard deviation, while those with a non-normal distribution were represented by median and interquartile range (IQR). Non-normally distributed continuous variables were compared between groups using non-parametric tests, and categorical variables were assessed using the chi-square test. The Kaplan-Meier (KM) method was used to calculate survival rates and compare the survival curves of the two groups, and test using Log Rank (Mantel-Cox). All statistical tests were performed using two-sided tests, and P < 0.05 was considered a statistically significant difference.

## Result

### Patient characteristics

**Figure 1:**
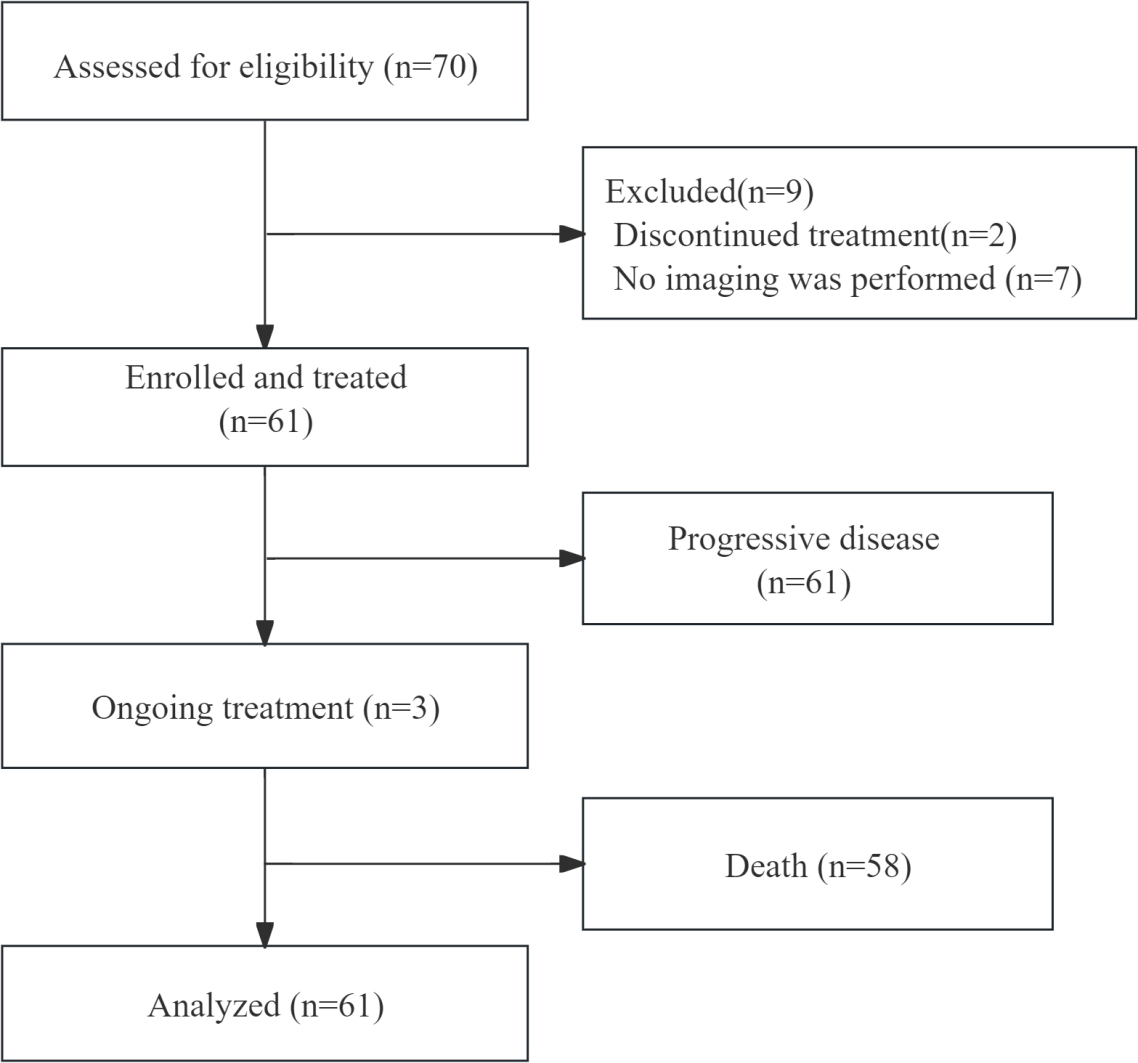
Flow chart of the selected population.

Between June 1, 2018, and June 30, 2021, a total of 70 patients were screened. All patients received 250 mg apatinib or 500 mg apatinib. On the data cutoff of June 31, 2022, 61 patients were included in the efficacy and safety analyses. 2 patients discontinued treatment without any imaging available for review; 7 patients could not undergo examination because of the COVID-19 outbreak, as shown in **Figure 1**. Baseline patient characteristics are summarized in **Table 1**.

**Table 1:** The characteristics of included patients.

Variables	All patients (N=61)	HDEI (N=28)	LDEI (N=33)	*P* value
Demographics				
Age (years), median (±SD)	63.61±9.26	63.07±9.54	64.06±9.14	0.6812
Male	39 (63.93%)	17 ( 60.71%)	22 ( 66.67%)	0.629
ECOG performance status				0.973
0	26 (42.62%)	12 (42.86%)	14 (42.42%)	
1	35 (57.38%)	16 (57.14 %)	19 (57.58%)	
Initial doses				0.004
250mg	34 (55.74%)	10 (35.71%)	24 (72.73%)	
500mg	27 (44.26%)	18 (64.29%)	9 (27.27%)	
Medication time				0.111
Long medication time	26 (42.62%)	15 (53.57%)	11 (33.33%)	
Short medication time	35 (57.38%)	13(46.43%)	22 (66.67%)	
Treatment line				0.680
1	16 (26.23%)	7 (25.00%)	9 (27.27%)	
2	27 (44.26%)	14 (50.00%)	13 (39.39%)	
3	18 (29.51)	7 (25.00%)	11 (33.33%)	
Combined immunotherapy				0.426
Yes	25 (40.98%)	13 (46.43%)	12 (36.36%)	
No	36 (59.02%)	15 (53.57%)	21 (63.64%)	
Combined Chinese Medicine				0.426
Yes	25 (40.98%)	13 (46.43%)	12 (36.36%)	
No	36 (59.02%)	15 (53.57%)	21 (63.64%)	
Stage				0.234
IV	29 (47.54%)	11 (39.29%)	18 (54.55%)	
III	32 (52.46%)	17 (60.71%)	15 (45.45%)	

SD, standard deviation; ECOG, Eastern Cooperative Oncology Group; HDEI, high-dose exposure intensity; LDEI, low-dose exposure intensity.

### Efficacy

#### Analysis of the relationship between dose exposure intensity and patient survival

**Figure 2:**
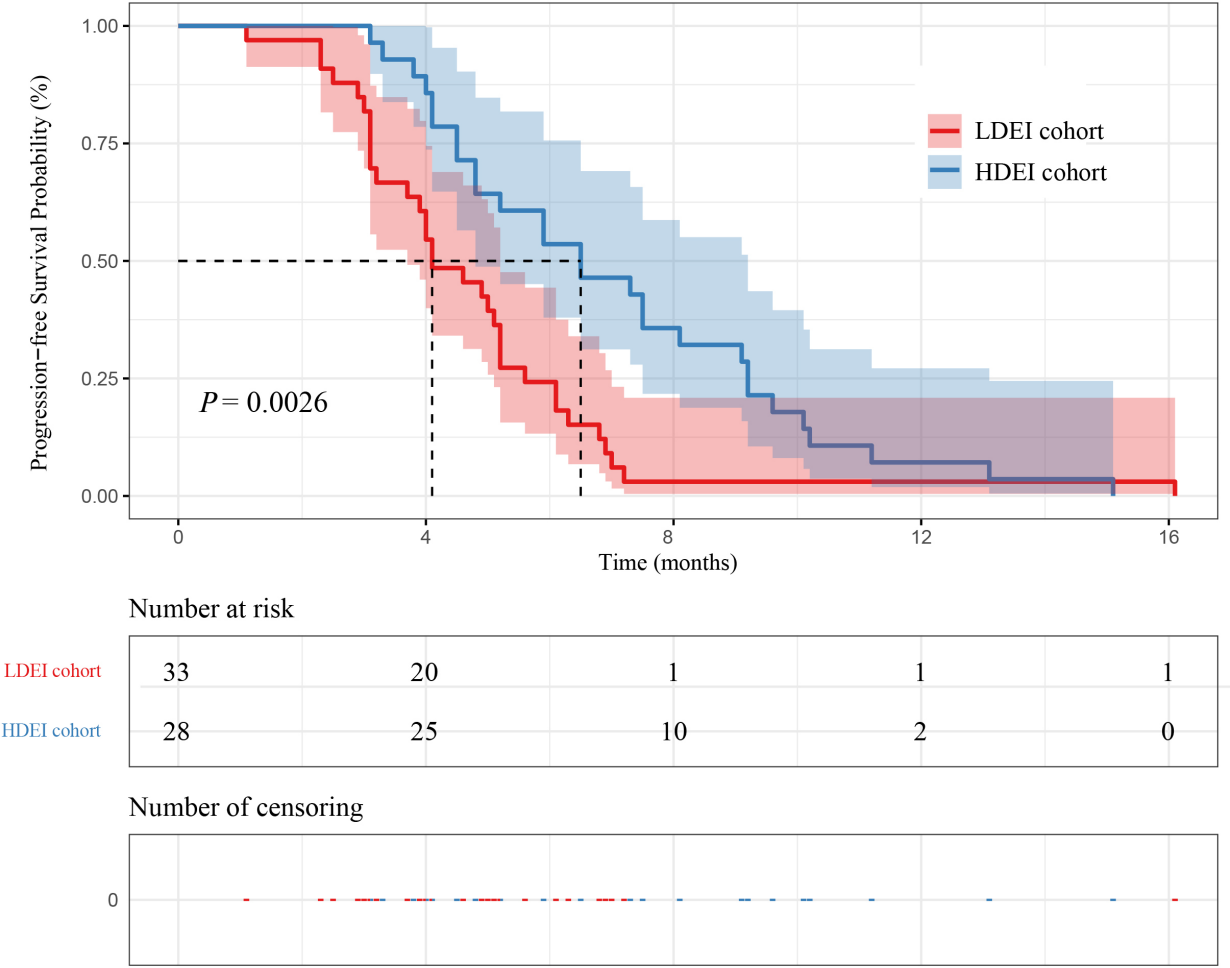
Kaplan–Meier curves for PFS between dose exposure intensity and patient survival.

**Figure 3:**
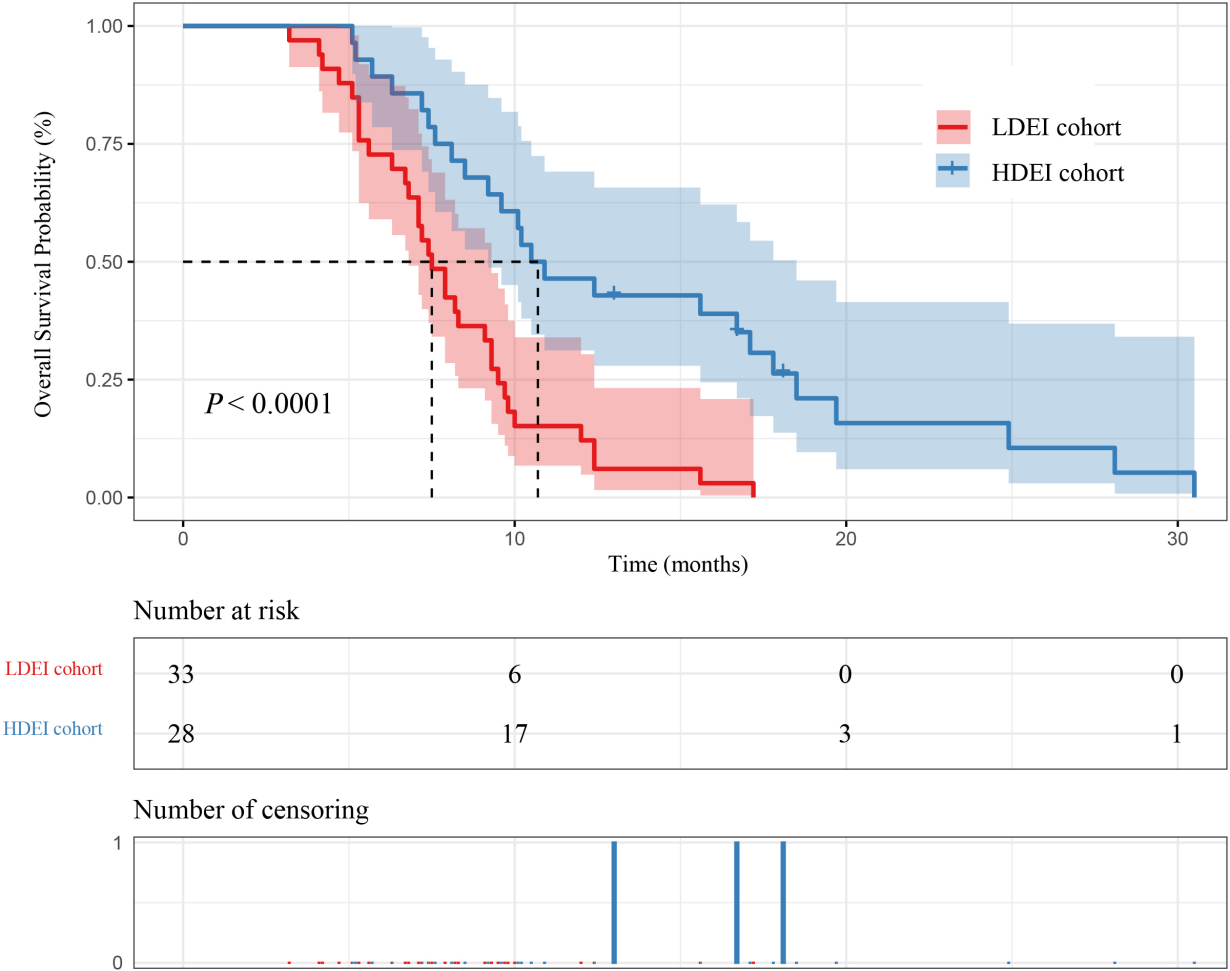
Kaplan–Meier curves for OS between dose exposure intensity and patient survival.

Among the 61 patients, 28 patients were in the HDEI cohort, and 33 were in the LDEI cohort. The median PFS (mPFS) of patients in the HDEI and the LDEI group were 6.50 months (95% confidence interval [4.80-9.20]) and 4.10 months (95% CI [3.70-5.20]) (P=0.0026), respectively. The median OS (mOS) of patients in the HDEI and the LDEI group were 10.70 months (95% CI [9.20-18.50]) and 7.50 months (95% CI [6.80-9.30]) (P<0.0001), respectively. The mPFS and mOS in the HDEI cohort was higher than the LDEI cohort, as shown in **Figures 2** and **3**.

### Analysis of the relationship between Initial doses and patient survival

**Figure 4:**
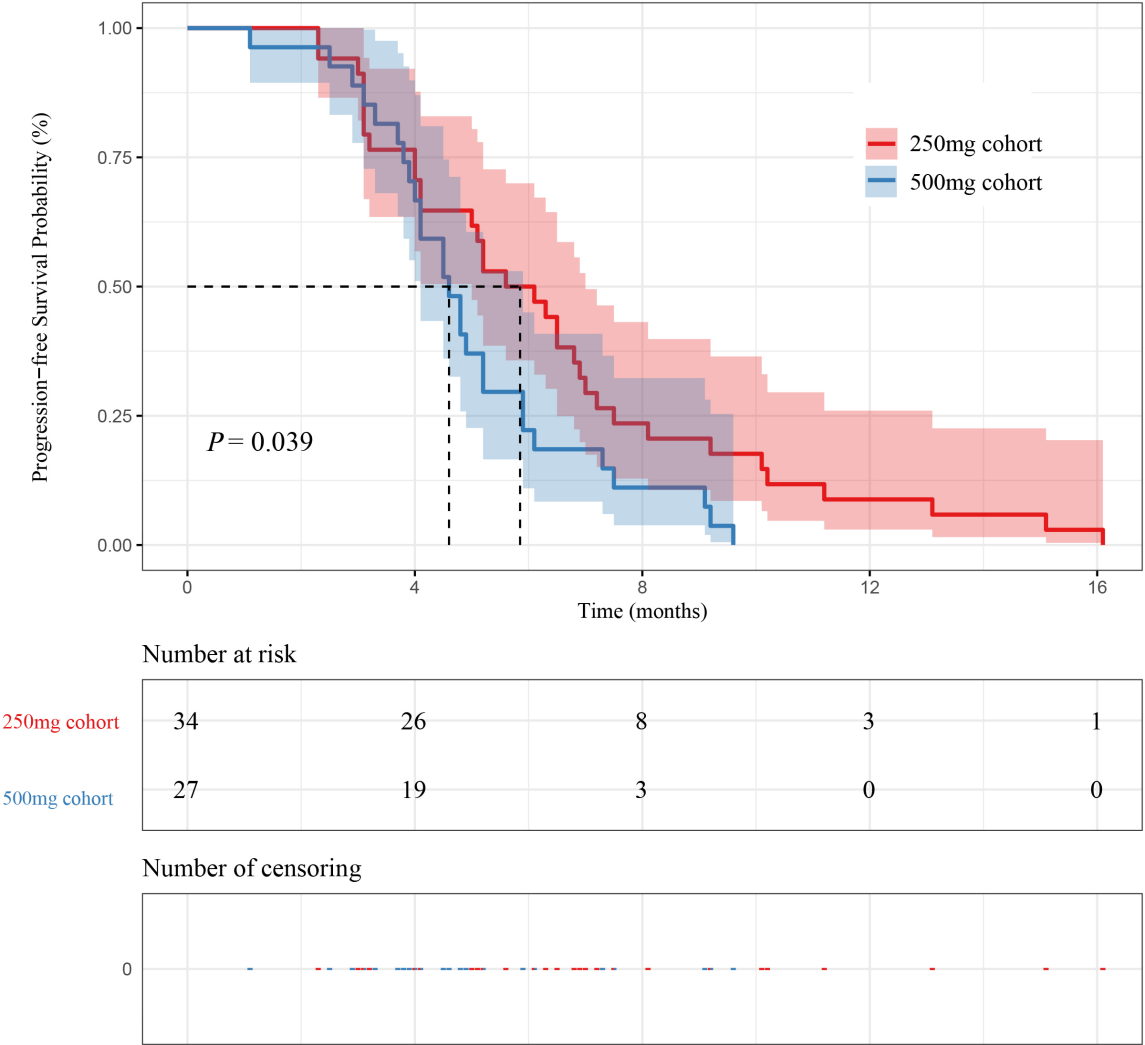
Kaplan–Meier curves for PFS between initial doses and patient survival.

**Figure 5:**
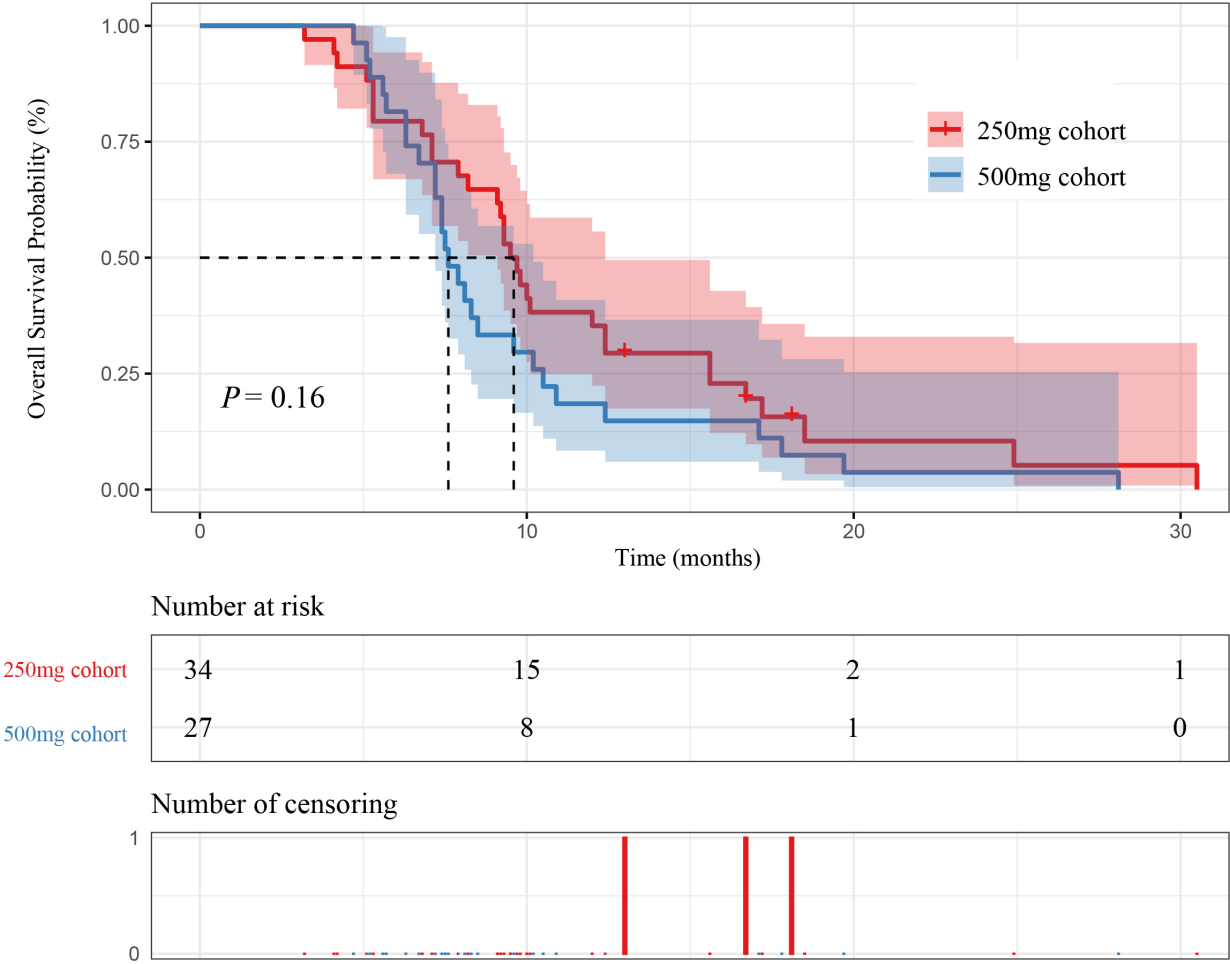
Kaplan–Meier curves for OS between initial doses and patient survival.

In 61 patients, 34 patients were in the 250 mg cohort, and 27 patients were in the 500 mg cohort. The mPFS of patients in the 250 mg cohort and the 500 mg cohort were 5.85 months (95% CI [5.00-7.00]) and 4.60 months (95% CI [4.10-5.90]) (P=0.039), respectively. The mOS of patients in the 250 mg cohort and the 500 mg cohort were 9.60 months (95% CI [9.10-12.40]) and 7.60 months (95% CI [7.20-10.20]) (P=0.16), respectively. The mPFS in the 250 mg cohort was higher than 500 mg cohort, as shown in **Figures 4** and **5**.

### Analysis of the relationship between medication time and patient survival

**Figure 6:**
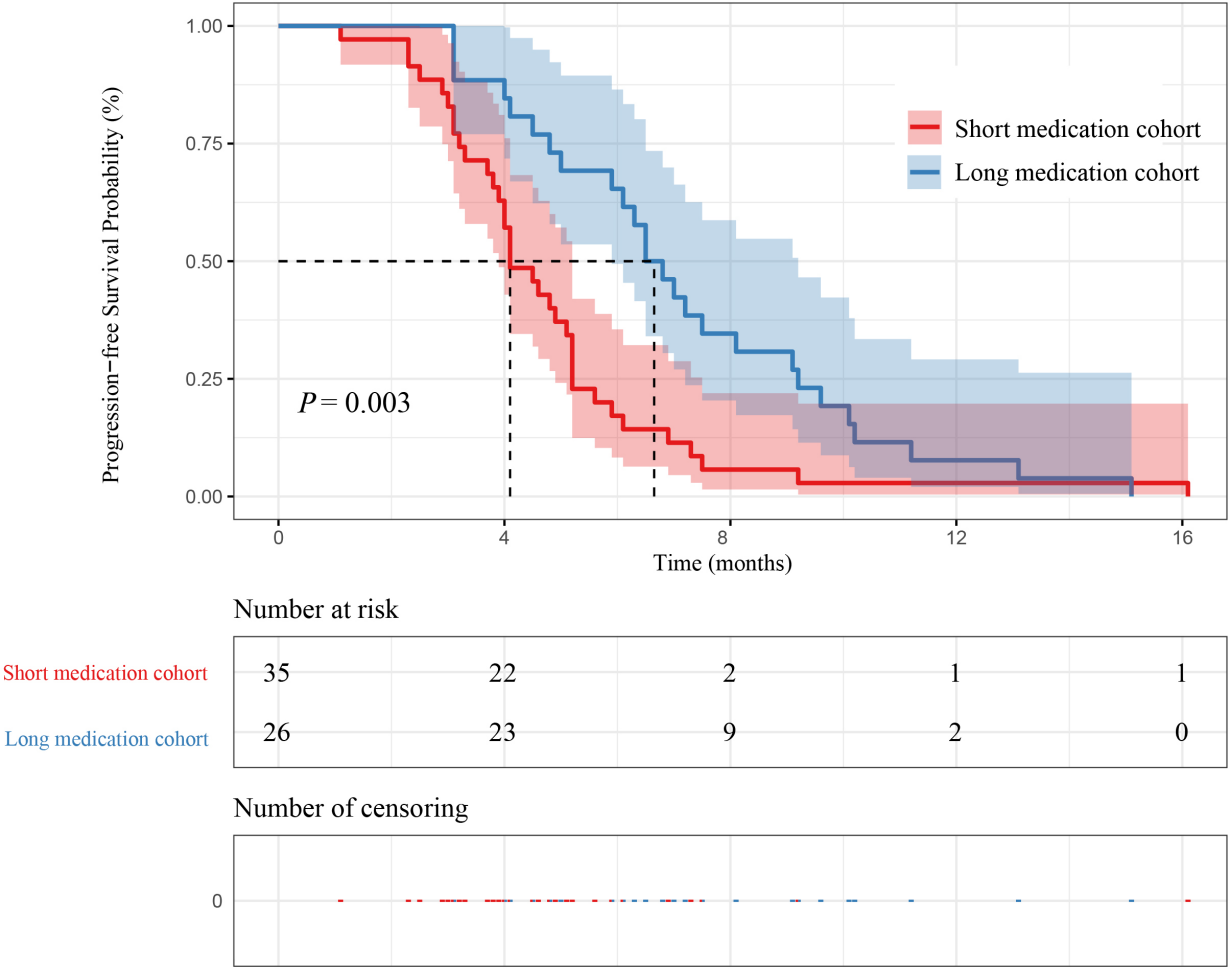
Kaplan–Meier curves for PFS between medication time and patient survival.

**Figure 7:**
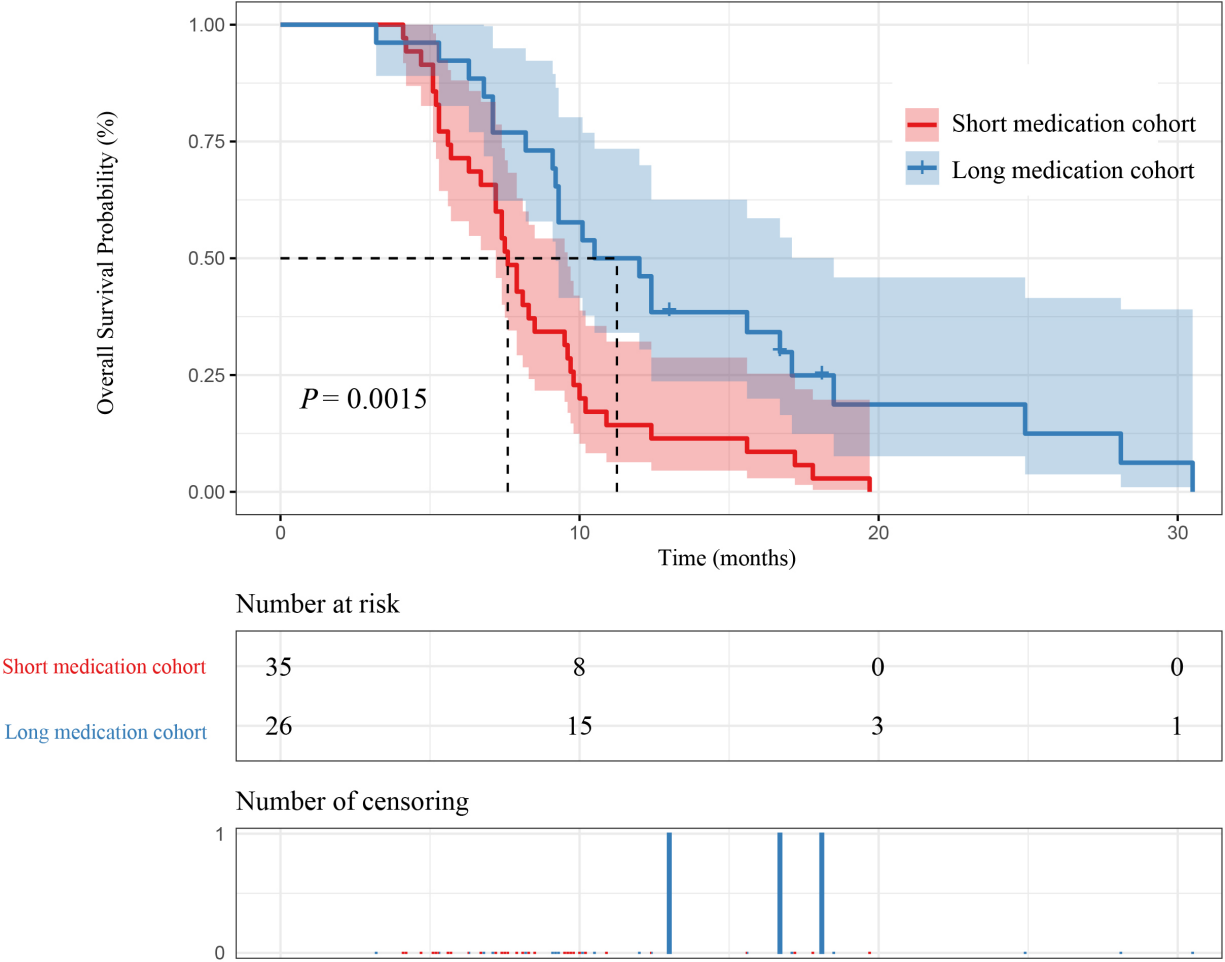
Kaplan–Meier curves for OS between medication time and patient survival.

In our study, 26 patients were in the long medication time cohort, and 35 patients were in the short medication time group cohort. The mPFS of patients in the long medication time cohort and the short medication time cohort were 6.65 months (95% CI [5.90-9.20]) and 4.10 months (95% CI [3.90-5.20]) (P=0.003), respectively. The mOS of patients in the long medication time cohort and the short medication time cohort were 11.20 months (95% CI [9.20-18.50]) and 7.60 months (95% CI [7.20-9.60]) (P=0.0015), respectively. In this case, the mPFS and mOS in the long medication time cohort was higher than short medication time cohort, as shown in **Figures 6** and **7**.

### Safety

All the 61 patients were included in the safety analysis, and treatment-related adverse events (TRAEs) were listed in **Table 2**. All the patients experienced at least 1 adverse reaction. The most common TRAEs of any grade were hypertension (17[27.87%]), proteinuria (14[22.95%]), and neutrophil count decreased (14[22.95%]). Grade ≥3 TRAEs were 4 cases in the 250 mg apatinib cohort (1 hypertension, 1 proteinuria, 1fatigue, 1 aspartate aminotransferase increased), 13 cases in the 500 mg apatinib cohort (3 hypertensions, 3 proteinuria, 3 abdominal pain, 2 diarrhea, 1 hand-foot syndromes, 1 neutrophil count decreased). The ≥3 TRAEs in the 500 mg cohort were higher than the 250 mg cohort (P=0.0016) (**Table 2**).

**Table 2:** Treatment-related adverse events (n=61).

Treatment-related adverse events, n (%)	All Grade (n=61)	Grade≥3 (n=21)		P value
		250 mg apatinib cohort	500 mg apatinib cohort	
Any event	61 (100.00)	4 (19.05)	13 (61.90)	*P*=0.0016
Hypertension	17 (27.87)	1 (4.76)	3 (14.29)	
hand-foot syndrome	9 (14.75)	0	1 (4.76)	
Proteinuria	14 (22.95)	1 (4.76)	3 (14.29)	
Diarrhea	8 (13.11)	0	2 (9.52)	
Emesis	3 (4.92)	0	0	
Nausea	7 (11.48)	0	0	
Abdominal pain	12 (19.67)	0	3 (14.29)	
Positive fecal occult blood test	4 ( 6.56)	0	0	
White blood cell count decreased	9 (14.75)	0	0	
Neutrophil count decreased	14 (22.95)	0	1 (4.76)	
Platelet count decreased	13 (21.31)	0	0	
Anemia	6 (9.84)	0	0	
Decreased appetite	9 (14.75)	0	0	
Fatigue	11 (18.03)	1 (4.76)	0	
Aspartate aminotransferase increased	9 (14.75)	1 (4.76)	0	
Alanine aminotransferase increased	8 (13.11)	0	0	
Gamma-glutamyltransferase increased	12 (19.67)	0	0	

## Discussion

In this observational, retrospective cohort study, low-dose apatinib provided clinically meaningful anti-tumor activity and a manageable safety profile in AGC. The mPFS and mOS were 6.50 and 10.70 months in HDEI cohort, respectively. The mPFS and mOS were 5.85 and 9.60 months in 250 mg cohort, respectively. The mPFS and mOS were 6.65 and 11.20 months in long medication time cohort, respectively. The grade 3 or greater treatment-related adverse events, especially those related to hematological and gastrointestinal toxicity, were less commonly reported in 250 mg cohort. Our study may shed light on dose exposure intensity may be a new indicator for evaluating drug efficacy, and 250 mg apatinib as a potential treatment option for patients with AGC.

In clinical studies, the daily dose is called “daily exposure.” However, this ignores the duration of medication, and this study introduces a concept of “dose exposure intensity.” Dose exposure intensity is the product of dose and duration of medication. The purpose of dose exposure intensity is to determine the efficacy and safety of apatinib 250 mg and 500 mg by evaluating the relationship between dose and medication time. In our study, HDEI significantly Improves PFS and OS in Patients with AGC. Although only a total of 35.71% of patients initiated apatinib at 250 mg in HDEI cohort, these patients were in long medication time cohort, indicating the long duration of low-dose apatinib contributes more to the HDEI. In a study about apatinib pharmacokinetic, it was found that increase in apatinib exposure was less than proportional to dose from 250 to 850 mg, and the relative bioavailability of apatinib will decrease as the dose increases (15). Therefore, the clinical efficacy of apatinib is not only related to the dose, but also to the medication duration. The mPFS and mOS in the long medication time was longer than short medication time cohort, indicating the dose exposure intensity is significantly positively correlated with the medication time. Prolonging the medication time of apatinib can increase the exposure dose of apatinib in the body. Therefore, the apatinib of HDEI can prolong PFS and OS, improving clinical efficacy in patients of AGC.

In the phase 3 clinical trial of apatinib, oral apatinib 850 mg improved patients’ PFS and OS (2.6 months and 6.5 months, respectively) (16). However, of the 40 patients that discontinued apatinib treatment, 22 patients (55%) stopped treatment as a result of toxicity, and dose reduction occurred in 21% patients who finished apatinib treatment. The remaining patients received a reduced dose of apatinib. However, specific data on the reduced dose and adverse effects are lacking (17). Therefore, the results of the Phase 3 clinical trial of apatinib remain uncertain, and it cannot be concluded that 850 mg of apatinib is safe and acceptable for patients with AGC. In several ongoing clinical trials, the initial dosage of apatinib was 250 mg or 500 mg (18–22). However, the article did not explain the reasons for using this dose in clinical trials. Currently, there are few studies on the dosage and pharmacokinetics of apatinib. In multiple-dose up-titration study of apatinib in AGC, apatinib exposure increased in a dose-dependent manner over the 500 mg to 850 mg dose range, and patients who were up-titrated to 850 mg had lower drug exposure than those who were not (23).Nevertheless, dose titration of 250 mg of apatinib was not performed in the article, and it is unclear whether apatinib exposure increases in a dose-dependent manner in the dose range of 250 mg to 500 mg. In a meta-analysis of the efficacy of low-dose apatinib in treating AGC, it shown that low-dose apatinib (250 mg or 500 mg) combined with chemotherapy as a second-line treatment improve the objective response rate (ORR), disease control rate (DCR), PFS and OS compared with chemotherapy alone (24).Despite the article did not compare the efficacy of 250 mg or 500 mg apatinib, it proved that low-dose apatinib can prolong patient survival without increasing adverse reactions in AGC.

Fluorouracil-based regimens combined with platinum or taxane drugs are first-line treatment options in AGC. In a study evaluating the XELOX regimen in AGC, the mPFS and mOS of the XELOX group were 5.0 months and 12.0 months, respectively (25). In a study of paclitaxel combined with capecitabine in AGC, the mPFS and mOS of the paclitaxel combined with capecitabine group were 5.0 months and 12.5 months, respectively (26). Paclitaxel combined with ramucirumab is the main second-line treatment for AGC, and the mPFS and mOS were 4.14 months and 8.71 months, respectively (27). In the third-line treatment of GC, nivolumab is recommended, with mPFS and mOS of 2.0 months and 5.90 months, respectively (28). In our study, the mPFS and mOS in the 250 mg apatinib group were 5.85 months and 9.60 months, respectively. Although more than 70% of the patients received second-line and third-line treatment, the results were comparable to the current standard treatment regimens and showed a significant advantage in improving PFS. This further confirms the significant efficacy of 250 mg apatinib in the treatment of AGC.

Although 850 mg is the recommended dose, it is rarely used in clinical practice. Current studies have demonstrated the efficacy and safety of 250 mg and 500 mg apatinib in the Clinic. However, there is a lack of comparative studies on the efficacy and safety of low-dose apatinib. In our study, compared with 500 mg apatinib, 250 mg apatinib increased the medication time and dose exposure intensity of apatinib, prolonged PFS in patients with AGC. Moreover, compared with standard treatment options, low-dose apatinib has significant advantages in improving PFS.

Hypertension, hand-foot syndrome, myelosuppression, and proteinuria are common TRAEs associated with VEGF/VEGFR inhibition, often reported in studies of angiogenesis inhibitors, such as apatinib (29), sorafenib (30), sunitinib (31), lenvatinib (32). In the safety study, the most common adverse reactions were hypertension, proteinuria, myelosuppression, which was consistent with the results of previous adverse reaction studies of apatinib, with no new safety signals identified (16, 33).The incidence of grade 3-4 adverse reactions in the 500 mg cohorts was significantly higher than that in the 250 mg cohorts, mainly including hypertension, proteinuria and abdominal pain, which was the reason for the patient discontinuing medication. Therefore, it is necessary to fully consider the trade-off between dose intensity, efficacy, and safety.

Furthermore, despite apatinib is cheaper than other anti-tumor drugs, the price of each 250mg apatinib tablet is $ 20, which is already unaffordable for many Chinese families. The economic burden of patients should be considered while considering the efficacy. There is currently no comparative study on the efficacy and safety of 250mg and 500mg apatinib. In the case of no significant difference in efficacy, can we consider reducing the economic burden of patients and using 250 mg apatinib?

To our knowledge, this is the first study to analyze dose exposure intensity and efficacy and safety of 250 mg and 500 mg apatinib. However, Several limitations of this study should be acknowledged. First, this study is a small sample, single-center retrospective cohort study, and data need to be interpreted in the context of historical comparison that may introduce bias. Second, this study was conducted during the COVID-19 pandemic, resulting in patients experiencing medication delays, which may lead to disease progression. Some patients fail to review on time, which may prolong the patient’s PFS to a certain extent. Considering the above factors, to improve the credibility and accuracy of the results, this study did not calculate the DCR and ORR of patients. Third, investigators assessed tumor response and there was no central review. Finally, our study is not a randomized controlled trial, and patients may have received other anti-tumor treatments while taking apatinib, which may affect the interpretation of the results. The results observed in this study should be further investigated and verified in a randomized controlled trial.

## Conclusion

The efficacy of apatinib is closely related to dose exposure intensity, medication time, and initial dose. Early application of 250mg apatinib can reduce drug adverse reactions and improve patient tolerance of the drug, thereby achieving longer drug exposure time. Therefore, 250mg apatinib can be used as the recommended dose for advanced gastric cancer.

## Data Availability

The raw data supporting the conclusions of this article will be made available by the authors, without undue reservation.
